# Life Extension Strategies of Wind Turbine Gearbox Based on Multi-Source Information Fusion Under Different Control Strategies

**DOI:** 10.3390/s26123759

**Published:** 2026-06-12

**Authors:** Yili Wang, Caichao Zhu, Xinhao Luo, Jianjun Tan

**Affiliations:** 1 School of Mechanical Engineering, Xihua University, Chengdu 610039, China; 2 The State Key Laboratory of Mechanical Transmission, Chongqing University, Chongqing 400044, China

**Keywords:** wind turbine gearbox, multi-source information fusion, remaining useful life prediction, life extension

## Abstract

Wind turbine gearbox failures lead to substantial downtime and high maintenance costs. Although condition-monitoring systems are widely used, traditional life-extension methods that simply reduce power output often decrease revenue. Current research frequently treats life optimization and power generation independently, and as such lacks a quantitative link between control strategies and remaining useful life. To address this gap, this paper proposes a novel life-extension strategy that optimizes power generation by dynamically adjusting rotor speed and pitch angle. A transfer learning–long short-term memory model enhanced by multi-source information fusion is developed to predict remaining useful life accurately under conditions with limited fault data. Utilizing real operational data from 2 MW wind turbines in Northeast China, the study quantitatively analyzes the impact of variable-speed and pitch control. The results demonstrate that while both strategies extend life, variable-speed control offers superior effectiveness in improving remaining useful life. Furthermore, maximum power generation is achieved not at full capacity, but when the output is reduced to approximately 70% of the nominal power. At this optimal point, the proposed strategy increases power generation by up to 7.3%. This establishes a dynamic balance between operational safety and economic efficiency, overcoming the limitations of conventional methods.

## 1. Introduction

Wind energy is a clean and renewable energy source, and it represents a major direction in the global transition toward green and low-carbon energy. For wind turbines intended for long-term operation, operation and maintenance costs may account for approximately 30% of total expenditure [1]. In addition, gearbox failures have the longest failure duration, leading to the greatest economic losses [2]. For this reason, many wind farms install condition-monitoring systems to provide early fault warnings before transmission failure occurs. After a warning signal is received, wind farms commonly repair gearboxes immediately, although the gearboxes may still be capable of operating at reduced output levels. Since wind farms are frequently located in remote areas, continuous maintenance for minor faults in an individual gearbox can substantially increase overall operation and maintenance costs. Therefore, accurate evaluation of the health status of wind turbine gearboxes (WTGs), effective prediction of their remaining useful life (RUL), and development of a scientific life-extension strategy are essential for improving operational efficiency, lowering operating costs, and increasing revenue.

Accurate knowledge of the RUL of the transmission chain is essential for evaluating its safety and economic feasibility after life-extension measures. In recent years, progress in big data technology has made data-driven RUL methods an important research focus, promoting the use of emerging technologies, including deep learning, virtual reality, and digital twins, for fault prediction [3,4,5] and RUL prediction [6,7,8] of WTGs. External environmental factors, including wind speed and ambient temperature, were integrated into analytical framework, allowing gearbox operating conditions to be assessed more comprehensively by considering environmental influences [9]. A new diagnostic method was developed to address the low computational efficiency of conventional fault diagnosis models [10]. Previous studies [11,12] have presented and discussed the results of induction generator and doubly fed induction generator systems under different fault conditions as a function of the static compensator.

Due to the imbalance of actual operating data and the limited availability of fault samples, researchers have proposed a method based on digital analog linkage that utilizes digital twin methods [13,14,15,16,17,18,19] to integrate mechanisms and data while improving diagnostic interpretability and reliability. In addition, to avoid information loss, scholars have proposed a method that applies visual and image information to research on health status assessment for the state detection of industrial machinery based on multimodal data [20,21,22,23,24,25,26].

At present, many wind turbines worldwide have reached the later stages of their service life, making life-extension research an emerging area of interest. Numerous studies have shown that life extension can substantially increase wind farm revenue. Previous work [27,28] has summarized recent advances in life extension for onshore wind turbines in European countries. One study [29] examined the effect of blade life extension on total power generation. Other researchers [30,31] have identified optimal solutions for offshore wind farm life-extension management from an economic perspective. Another study [32] proposed a framework based on two extreme scenarios to evaluate the technical and economic feasibility of wind farm life extension, whereas another researcher [33] performed a technical and economic analysis of wind farm life extension.

These studies mainly emphasize macro-level assessments of feasibility and economic effects, while specific methods for extending the life of wind turbines remain insufficiently addressed. Consequently, considerable gaps persist in practical engineering applications, and limited research has examined concrete life-extension methods. One study [34] proposed a gearbox life-extension method based on reducing the power output of wind turbines, while another [35] introduced an adaptive RUL control strategy for blades. Another researcher [36] performed a life-extension analysis of wind turbine blades and improved model accuracy using a particle swarm optimization (PSO) algorithm.

Overall, current research on wind turbine life extension remains at an early stage, mainly concentrating on macro-level feasibility and economic aspects while seldom examining the effects of life-extension strategies on RUL. The relationship between life-extension methods and RUL has rarely been quantified, and this separation can readily create challenges when applying the resulting strategies in practical engineering applications.

To address these issues, this study proposed a practical life-extension strategy involving adjustment of the speed and pitch angle of WTGs and quantified how different control strategies affect the RUL of WTGs. The innovation of this study lies in establishing a quantitative relationship between the RUL of WTGs and wind turbine power generation. A dynamic balance between RUL and power generation is achieved, which overcomes the inherent limitation of traditional approaches that treat RUL and power generation as separate metrics. Consequently, this research enables simultaneous optimization of the operational safety and economic efficiency of wind turbine systems.

The rest of this paper is structured as follows. In Section 2, the methods used in this study are presented, including the transfer learning (TL) and long short-term memory (LSTM) model for calculating RUL, life-extension approaches, and optimization methods. In Section 3, the effects of life extension on RUL and power generation are discussed. In Section 4, the paper is summarized, and future work in this area is outlined.

## 2. Methods

**Figure 1:**
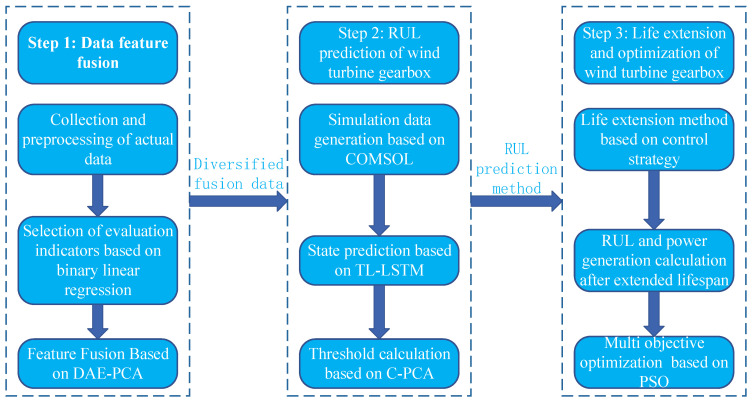
Flowchart of the method proposed in this paper.

The methodological workflow used in this study is as follows. First, to address noise and high-dimensional data, the denoising autoencoder and principal component analysis (DAE-PCA) method was employed for feature fusion using actual supervisory control and data acquisition (SCADA) data from 2 MW WTGs located in three wind farms in Northeast China. The extracted robust features were subsequently used to construct a transfer learning–long short-term memory (TL-LSTM) model for RUL prediction. The source-domain data, consisting of simulated WTG states generated using COMSOL software version 6.0, were first used to pre-train the LSTM model, after which the model was fine-tuned with the target-domain data, consisting of actual operational data, using maximum mean discrepancy (MMD) for domain adaptation. In addition, the limits of the evaluation indicators obtained from the SCADA system were combined with interval principal component analysis (C-PCA) to calculate the RUL limit. The RUL was then determined by comparing the model predictions with this threshold. Finally, a PSO algorithm was applied to achieve life extension while optimizing power generation. Based on the RUL of the WTG, the optimal control parameters were identified to maximize overall power generation while extending RUL. The flowchart of the entire method is shown in Figure 1.

To ensure the feasibility of the method, we validated the results of several key steps. In the evaluation indicator selection section, we presented the results of binary nonlinear correlation analyses for selected parameters. In the COMSOL software section, we demonstrated the deviation between the data generated by the software and the actual data. In the RUL prediction section, we validated the RUL prediction results for two WTGs. In the life extension and power generation section, we validated the RUL and power generation results using data from actual operating WTGs. The final optimization part of the life extension method was validated using data from multiple wind farms. A previous study [34] analyzed a life-extension strategy using a similar method and demonstrated the feasibility of the process.

### 2.1. Data Preprocessing

The example operating data analyzed in this paper were obtained from actual operating records of 2 MW wind turbines in three wind farms, with each wind farm consisting of 24 wind turbines, and the sampling interval was 1 min. Data from the first wind farm were collected from 16 January to 6 March 2017. Data from the second wind farm were collected from 30 December 2019 to 28 March 2020. Data from the third wind farm were collected from 25 May to 22 July 2020. The types of signals recorded in WTG mainly included the following:

1. Temperature signals: These signals included gearbox oil sump temperature, gearbox inlet oil temperature, and the temperature at the rear end of the high-speed shaft (HSS) of the gearbox.
2. Environmental signals: These signals included ambient temperature and wind speed, among other variables, and were typically used as external excitations for gearboxes.
3. Other signals: These signals were generally used as external excitations for gearboxes, including ambient temperature and wind speed.

Scattered points located far from the actual power curve were removed to obtain ideal training samples. Subsequently, the correlation between each signal was analyzed using binary nonlinear regression, and the correlations within this data subset are presented in Table 1. The analyzed signals included the rear bearing temperature of the high-speed shaft (*T_rb_*), spindle speed (*n*), generator torque (*T*), generator inlet oil pressure (*F_i_*), gearbox outlet pressure (*F_o_*), and gearbox oil temperature (*T_o_*). Table 1 shows that some signals have very high correlations, indicating that the information contained in these signals is similar; therefore, their simultaneous selection as predictors should be avoided. After the correlation analysis, a subset of SCADA signals was selected as the gearbox state evaluation indicators, as shown in Table 2.

In addition, a set of signals was selected as prediction signals to estimate the evaluation indicators. Considering prediction accuracy and model-training difficulty, the signals obtained in this study, which were mainly affected by external excitation, are summarized in Table 3. Data quality determines the accuracy and generalizability of the model. Actual data, however, often contain many missing values and noise, making them unsuitable for direct model training. Therefore, the obtained data must be screened and standardized. After standardization and cleaning, the DAE-PCA method was used to perform feature fusion on the multi-source gearbox data.

**Figure 2:**
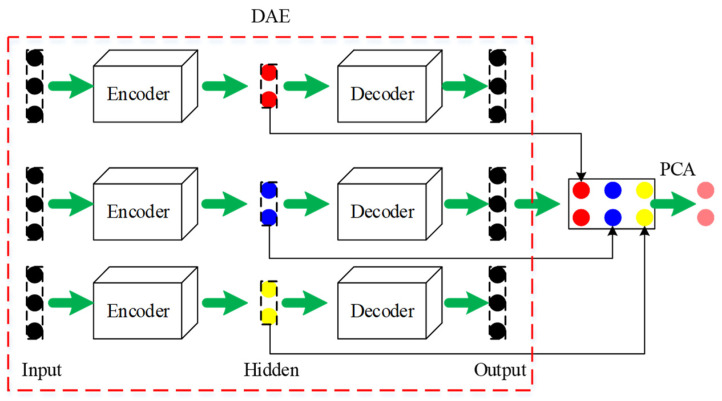
Feature fusion using the denoising autoencoder and principal component analysis (DAE-PCA) method.

Figure 2 presents the DAE-PCA structure. Raw data are first input to multiple parallel DAEs, where each DAE encodes noise-contaminated data to learn robust features in a hidden space and then decodes these features to reconstruct the original data, thereby verifying the validity of the extracted features. Subsequently, the hidden features from all DAEs are fed into the PCA module, which applies a linear transformation to extract the principal components, further reducing dimensionality while preserving the main variations in the data. This model is effective in noise filtering and high-dimensional data compression and is widely used in data preprocessing for tasks including image denoising, anomaly detection, and high-dimensional data visualization.

### 2.2. RUL Prediction Model

Given the limited amount of end-of-life data in the actual operating records of WTGs, it is necessary to augment the dataset with simulated data. TL can effectively address the problem of small fault samples in gearboxes. In this study, TL was integrated with the LSTM network to develop a state prediction model for a WTG, while interval-valued PCA was used to determine the RUL threshold.

**Figure 3:**
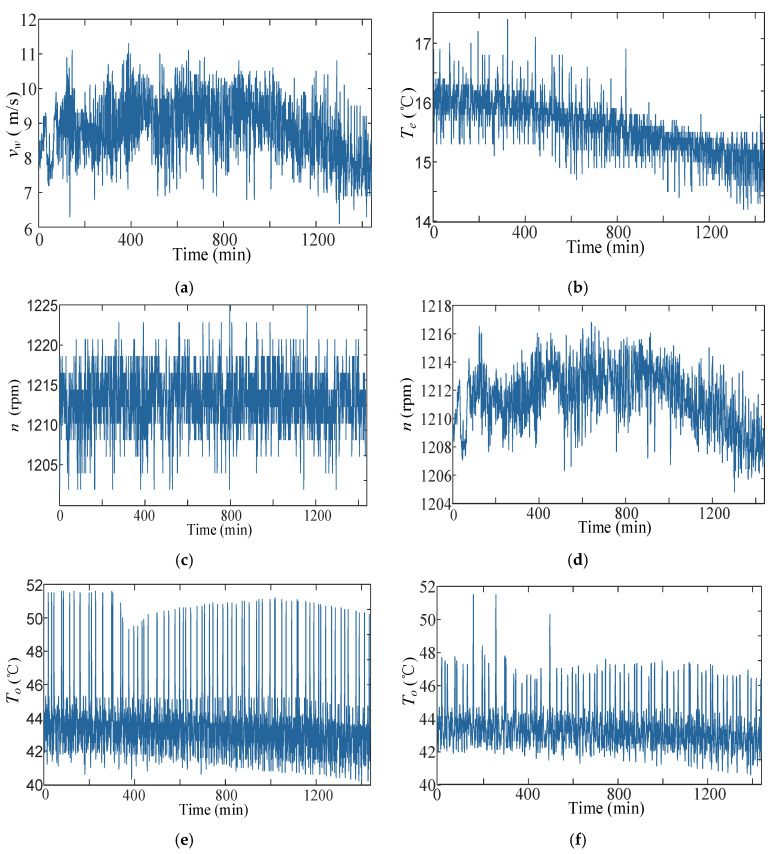
Differences between actual and simulated values. (**a**) Wind speed; (**b**) Environment temperature; (**c**) Actual rotational speed; (**d**) Simulated rotational speed; (**e**) Actual gearbox oil temperature; (**f**) Simulated gearbox oil temperature.

In this context, simulated data generated using COMSOL software were employed to increase the sample size, thereby improving the model’s recognition capability and generalization performance under small-data conditions. To further refine the analysis, a comparative study of simulated and real-world data was carried out, and the corresponding results are presented in Figure 3. Specifically, Figure 3a and Figure 3b show the wind speed and ambient temperature during WTG operation, respectively. Figure 3c and Figure 3d present the simulated and actual motor speed values, respectively; whereas Figure 3e and Figure 3f show the actual and simulated gearbox oil temperatures, respectively. Figure 3d,f indicate that the simulated curves are smoother, with very few abrupt fluctuations, and that their signal amplitudes are clearly lower than those of the measured data, which may be attributed to the presence of noise. Table 4 summarizes the key time-domain characteristics of the measured and simulated signals. The results in Table 4 show that the root mean square (RMS) values of the actual and simulated data are almost identical. A substantial difference in kurtosis is nonetheless observed, indicating that the actual system is subjected to more severe impact loads.

**Figure 4:**
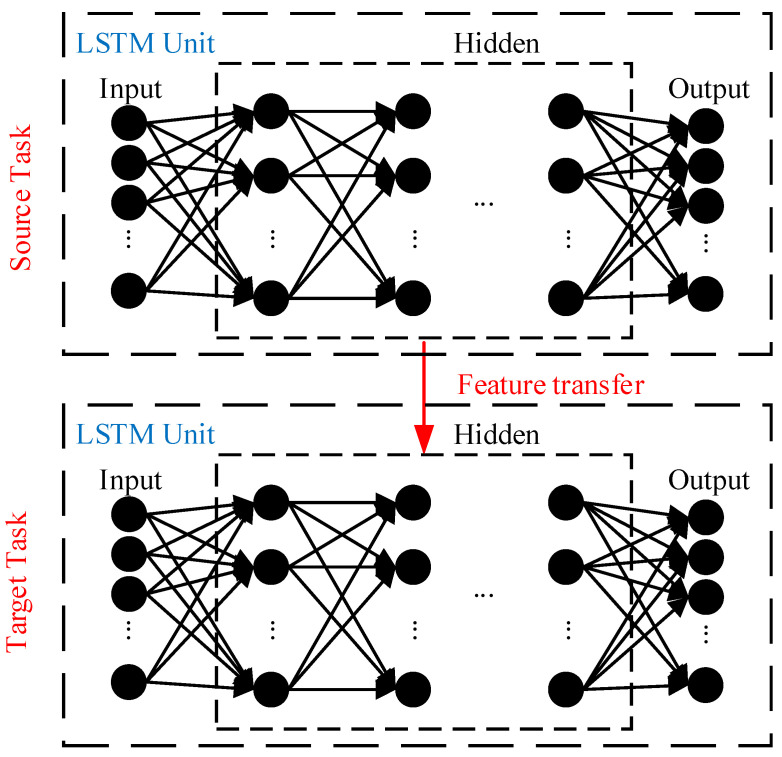
Structure of transfer learning–long short-term memory (TL-LSTM).

By using MMD to align the source-domain and target-domain data, an LSTM model was constructed using sufficient data from the source task. The features extracted from the source task were transferred to the target-task model through TL. The target-task data were then used to train the model through MMD. The dataset was divided into training and test sets at a ratio of 7:3. The structure of the TL-LSTM is shown in Figure 4.

Table 5 presents the limits of the evaluation index in the SCADA system. These limits are embedded in the SCADA system, and the wind turbine automatically shuts down once the evaluation indicators reach the threshold. Therefore, the RUL threshold can be calculated using the interval PCA method based on these limits.

For sample *x*, the midpoint matrix is calculated as follows:

(1) $$x^{c}=\left[\begin{array}{cccc} x_{11}^{c} & x_{12}^{c} & \ldots & x_{1n}^{c}\\ x_{21}^{c} & x_{22}^{c} & \ldots & x_{2n}^{c}\\ \vdots & \vdots & \ddots & \vdots \\ x_{m1}^{c} & x_{m2}^{c} & \ldots & x_{mn}^{c} \end{array}\right],$$

where $x_{ij}^{c}=(\max(x_{ij})+\min(x_{ij}))/2$ represents the median of the *j*th parameter in the *i*th vector.

The midpoint principal components can be obtained by applying PCA to the matrix *x^c^*:

(2) $$y_{ik}^{c}=\sum_{j=1}^{m}x_{ij}^{c}v_{jk}\;k=1,2,\ldots ,m,$$

where the value of parameter *j* on the *k*th principal component is denoted by $y_{ik}^{c}$, with *v_jk_* representing the *k*th eigenvector of *K^c^,* which is a hypermatrix in an *n*-dimensional vector space composed of all interval observation vectors.

The threshold for each principal component can be expressed as follows:

(3) $$y_{ik}^{-}=\sum_{j=1}^{m}\underset{\underline{x_{ij}}\leq x_{ij}\leq \bar{x_{ij}}}{\min}(x_{ij}v_{jk}),$$

(4) $$y_{ik}^{+}=\sum_{j=1}^{m}\underset{\underline{x_{ij}}\leq x_{ij}\leq \bar{x_{ij}}}{\max}(x_{ij}v_{jk}).$$

The RUL of the WTG can be effectively calculated by comparing the state predicted by the TL-LSTM model with the threshold obtained using the method described above.

### 2.3. Life-Extension Strategy Under Different Control Strategies

A previous study [37] indicated that the RUL of WTGs decreases as the load and speed increase. Therefore, lowering the output power of a WTG can reduce its load and support the objective of extending its RUL. At present, wind farms adopt two different control strategies to modify the operating state of wind turbines, thereby reducing the output power of WTGs.

**Figure 5:**
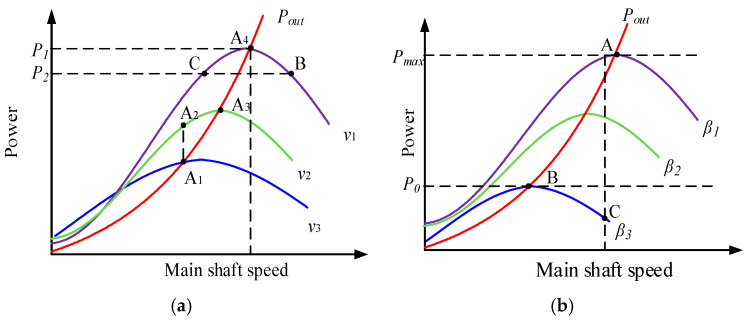
Principle for a wind turbine. (**a**) Variable-speed control; (**b**) Pitch control.

1. Variable-speed control: Figure 5a shows the relationship between output power and main shaft speed under different wind speeds. The *P_out_* curve represents the optimal output power, while the purple, green, and blue curves show the changes in output power with the main shaft speed at wind speeds of *v*_1_, *v*_2_, and *v*_3_, respectively, where *v*_1_ *> v*_2_ > *v*_3_. When the wind turbine operates at point A_1_, an increase in wind speed from *v*_3_ to *v*_2_ causes the aerodynamic power to increase suddenly, and the operating point of the wind turbine shifts to point A_2_. However, the main shaft speed cannot change abruptly because of inertia; therefore, the output power remains unchanged. Subsequently, since the output power is lower than the aerodynamic power, the main shaft speed gradually increases until the aerodynamic power becomes equal to the output power; in this case, point A_3_ becomes the new operating point of the wind turbine at a wind speed of *v*_2_. Consequently, when the wind speed and pitch angle remain unchanged, reducing or increasing the main shaft speed of the wind turbine can decrease the output power.
2. Pitch control: Figure 5b shows the relationship between output power and main shaft rotational speed under the same wind speed for different pitch angles. The *P_out_* curve represents the output power. The purple, green, and blue curves correspond to the power characteristics at different pitch angles, where *β*_1_ < *β*_2_ < *β*_3_. Figure 5 indicates that, when the wind speed remains constant, power reduction can be achieved by increasing the pitch angle.

### 2.4. Optimization Method for Life-Extension Strategy

**Figure 6:**
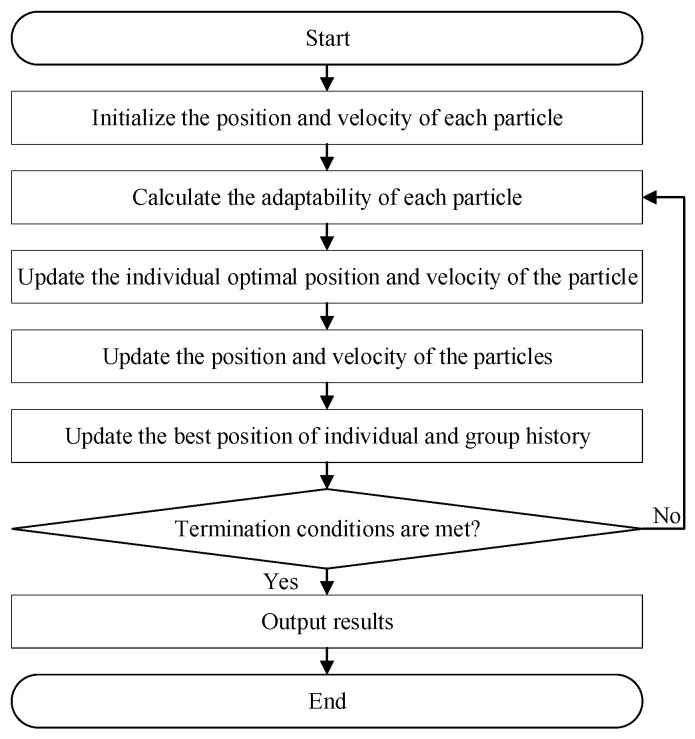
Flowchart of the PSO process.

As with typical industrial machinery, the economic status of a wind turbine must be evaluated after life extension. When the operation and maintenance policy remains unchanged, the primary factor influencing economic efficiency of a wind turbine before and after power reduction is the change in power generation [38]. Therefore, it is necessary to optimize the life-extension strategy based on power generation. In this paper, power generation is calculated according to the IEC 61400-12-1 standard [39], and the PSO algorithm is used to optimize the life-extension strategy. Figure 6 presents the flowchart of the PSO process, and the process steps are summarized as follows:

Step 1: Initialize the position and speed of each particle, along with the individual optimal position and global optimal position.

Step 2: Calculate the adaptability of each particle.

Step 3: If the fitness value of the current particle is higher than that of its individual optimal solution, update the individual optimal position and velocity of the particle.

Step 4: If the fitness value of the current particle is greater than that of the global optimal solution, update the global optimal position.

Step 5: Update the positions and velocities of all particles.

Step 6: Determine whether the termination condition is satisfied, such as whether the number of iterations has reached the predefined maximum or whether the global optimal solution has converged. If the termination criterion is met, terminate the algorithm; otherwise, return to Step 2 and continue the loop.

After training and calibration, this study sets the maximization of power generation as the objective. The maximum number of iterations is set to 1000, and the inertia factor is set to 0.8. Both the self-learning factor and the social learning factor are assigned a value of 3. The initial population size is 50, and the particle velocity bounds are set between −1 and 1.

## 3. Results and Discussion

### 3.1. Accuracy of the RUL Prediction Model

**Figure 7:**
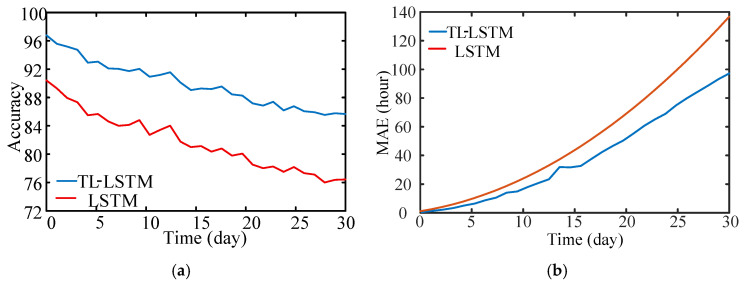
RUL prediction results. (**a**) Accuracy; (**b**) Mean absolute error.

The accuracy of the RUL prediction model had to be validated before discussing the implications of RUL prediction for maintenance. First, the RUL of 100 WTGs within 30 days was predicted. According to the RUL duration of the WTGs, they can be divided into 30 groups. The RUL of the *i*th WTG group is (*i* − 1, *i*) days. Figure 7a shows the accuracy of RUL prediction, which is calculated using the following equation:

(5) $$\eta =1-\frac{yp-ya}{ya},$$

where *y_p_* represents the predicted RUL, whereas *y_a_* represents the actual RUL.

Figure 7b presents the mean absolute error of RUL prediction. The results indicate that prediction accuracy improves after incorporating the TL module. In particular, prediction accuracy decreases as the prediction horizon becomes longer, since the influencing factors continue to increase. Nevertheless, the proposed method achieves an accuracy greater than 86%, which is comparable to the findings of previous research [40], thereby confirming its suitability for RUL prediction.

**Figure 8:**
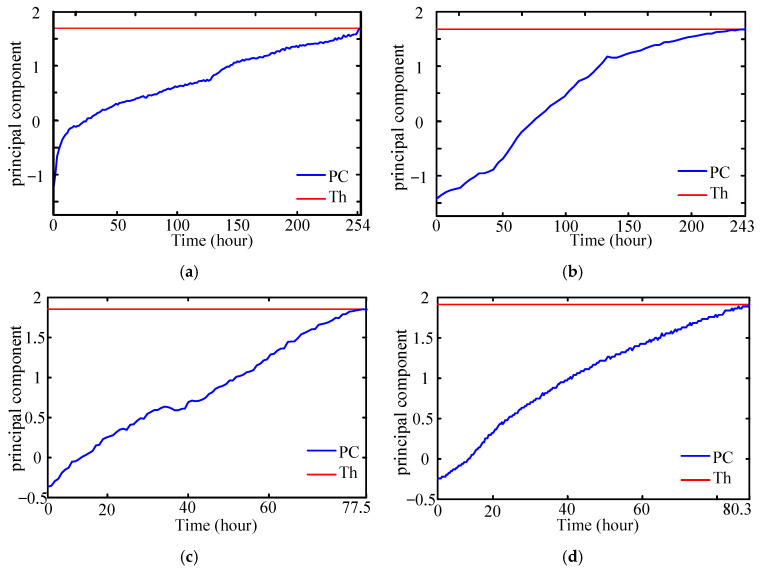
RUL prediction for WTGs. (**a**) First WTG using TL-LSTM; (**b**) First WTG using LSTM; (**c**) Second WTG using TL-LSTM; (**d**) Second WTG using LSTM.

Two specific WTGs were selected for analysis, as shown in Figure 8, where PC represents the principal component calculated using the TL-LSTM and PCA methods, while *Th* represents the threshold calculated using the C-PCA method. As shown in Figure 8, for the first WTG, the predicted RUL values using TL-LSTM and LSTM were 254 and 243.4 h, respectively. For the second WTG, the predicted RUL values using TL-LSTM and LSTM were 77.5 and 80.3 h, respectively. After reviewing the fault log, the actual RUL values of the first and second WTG were 261.2 and 73.1 h, respectively. These results further demonstrate that incorporating the TL module can improve the accuracy of RUL prediction.

### 3.2. RUL of WTGs After Life-Extension

**Figure 9:**
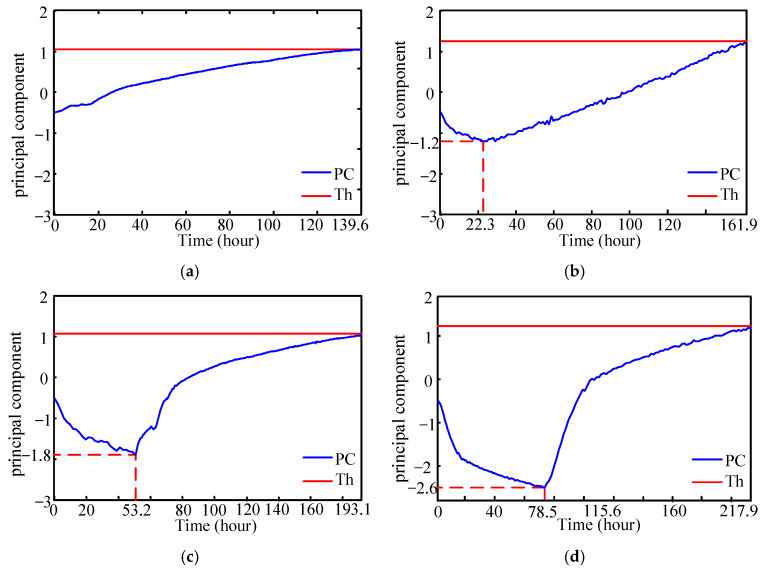
RUL of WTG under variable-speed control. (**a**) 100% power; (**b**) 90% power; (**c**) 80% power; (**d**) 70% power.

1. Variable-speed control: Under stochastic wind-speed fluctuations, adjusting the spindle speed to reduce power helps maintain the operational stability of the WTG after power reduction. Figure 9 shows the variation in the RUL of the WTG after life extension when variable-speed control is applied at a spindle speed of 1200 rpm. The simulation results show that the baseline RUL of the WTG is 139.6 h. When variable-speed control is used to reduce the power to 90%, 80%, and 70% of the nominal power, the RUL increases to 161.9, 193.1, and 217.9 h, respectively. The improvement in RUL is more evident in the medium-speed range than in the low-speed stage, since the change in the operating state of the WTG before and after power reduction is greater at medium speeds.

In addition, Figure 9 shows that during the degradation stage of the WTG, the performance of its core components initially decreases and then shows an upward trend. This phenomenon occurs because, after power reduction, the WTG gradually shifts into a low-power operating mode, and its performance then progressively deteriorates as faults develop.

**Figure 10:**
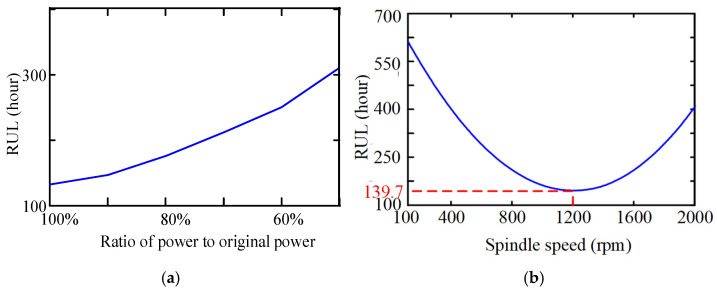
Changes in RUL of the WTG under variable-speed control. (**a**) With power; (**b**) Spindle speed.

As shown in Figure 10a, the RUL of the WTG changed when the actual power decreased to 50% of the original power. As the actual power of the WTG declined, its RUL gradually increased, and the rate of increase also progressively rose.

Figure 10b presents the variation in the RUL of the WTG with spindle speed. The graph clearly indicates that speed has a substantial effect on RUL. When the spindle speed was approximately 1200 rpm, the power of the WTG reached its highest level, while its RUL reached the lowest value of 139.7 h. It is evident that when the spindle speed decreased or increased, the power of the WTG gradually declined, while its RUL gradually increased. When the spindle speed increased to the cut-out speed, the RUL of the WTG was approximately 400 h. When the spindle speed decreased to 100 rpm, the RUL reached its maximum value.

**Figure 11:**
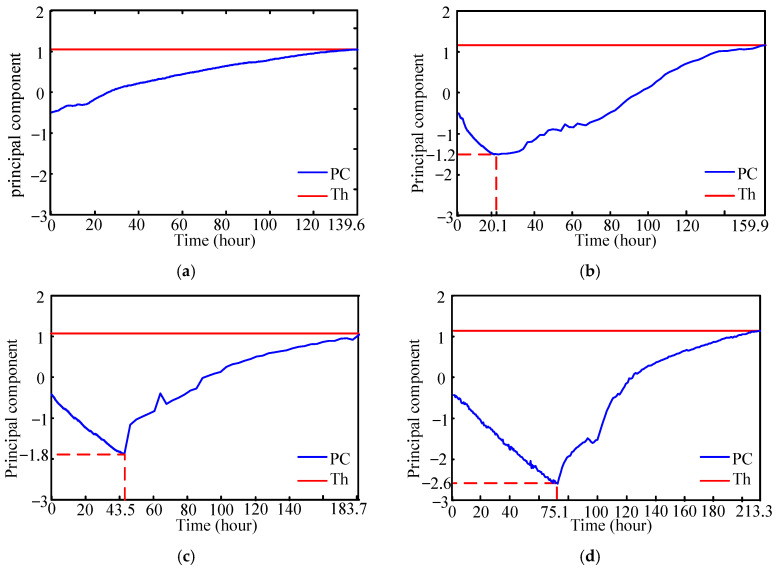
RUL of WTG under pitch control. (**a**) 100% power; (**b**) 90% power; (**c**) 80% power; (**d**) 70% power.

2. Pitch control: Figure 11 shows the RUL of the same WTG after life extension when pitch control is considered. The data indicate that the baseline RUL of the WTG is 139.6 h. When the power is reduced to 90%, 80%, and 70% of the nominal power, the RUL increases to 159.9, 183.7, and 213.3 h, respectively. Compared with variable-speed control, pitch control improves RUL by lowering power. This occurs because the pitch system of wind turbines is mechanically controlled, which leads to pitch delay and extends the operating duration under high-power conditions. The graph clearly shows that, in terms of principal component changes, all curves first decline and then rise, although the time required to achieve the same degree of decline is shorter.

**Figure 12:**
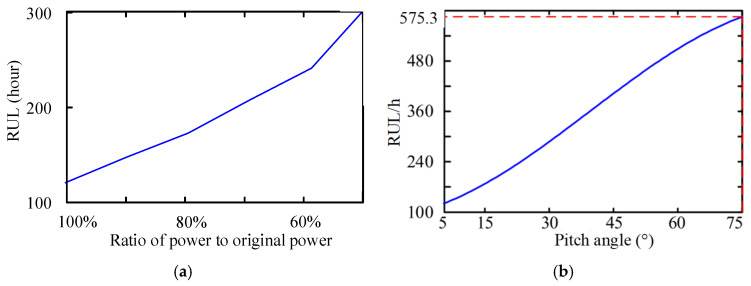
Changes in the RUL of WTG under pitch control. (**a**) With power; (**b**) With pitch control.

The variation in the RUL of the WTG under 50% power reduction is shown in Figure 12a. The graph indicates an inverse relationship between the output power of the WTG and its RUL: as the output power decreases, the RUL progressively increases, and the magnitude of this increase also becomes larger. Nevertheless, compared with variable-speed control, the improvement in RUL achieved through pitch control is relatively smaller.

Figure 12b shows the variation in RUL with respect to the pitch angle. The results indicate that the pitch angle has a more significant effect on RUL than power reduction alone. Specifically, the RUL of the WTG increases as the pitch angle rises. When the pitch angle reaches 75°, the RUL extends to approximately 575.3 h.

### 3.3. Optimization of Life-Extension Strategy for WTGs

The preceding analysis indicates that variable-speed control should be selected in both the medium- and high-speed stages when RUL is considered. However, due to the lag associated with pitch control, the average operating power of wind turbines is higher when pitch control is adopted. Consequently, when power generation is considered, the two strategies must be compared and analyzed.

**Figure 13:**
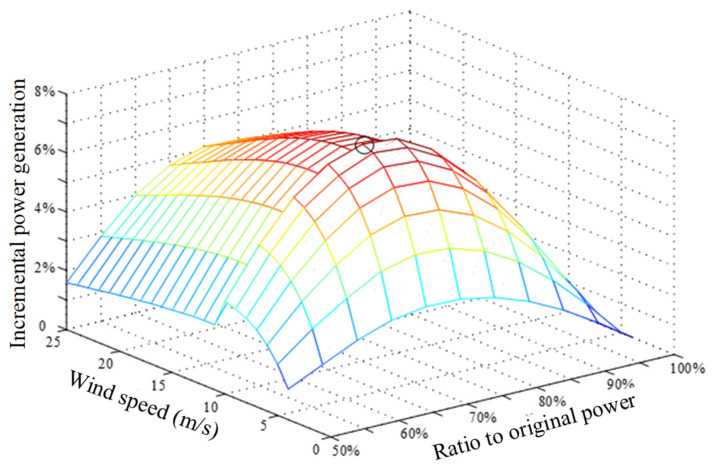
Power generation under variable-speed control.

1. Variable-speed control: Figure 13 shows the power generation achieved using variable-speed control. The results indicate that wind turbine power generation increases after life extension. A comparison of different power levels shows that power generation increases as output power is reduced until the actual power of the WTG reaches approximately 70% of its original capacity. At this point, power generation reaches its maximum value and then decreases with further power reduction. Regarding different wind speeds, power generation increases with wind speed until the rated wind speed is reached. Specifically, power generation reaches its maximum at a wind speed of 10 m/s and then decreases as the wind speed increases further. A possible reason is that 10 m/s is the rated wind speed of the analyzed wind turbine. The unit reaches its maximum power output at 10 m/s. When the wind speed exceeds this value, equipment safety requirements and design constraints cause the control system to limit further power growth, resulting in no additional increase in total power generation and even a slight decrease under the studied control strategy. Therefore, the occurrence of maximum power generation at 10 m/s is an inevitable outcome of the combined effects of the rated power characteristics of the wind turbine and the active power control strategy.

**Figure 14:**
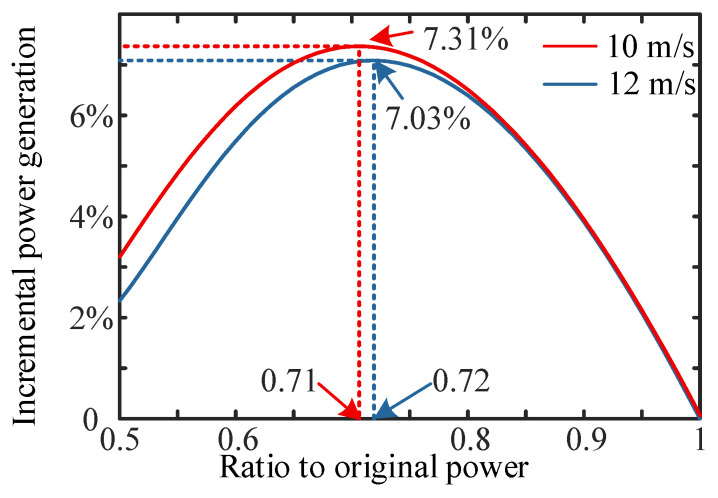
Power generation under variable-speed control at different power levels.

Figure 14 presents the differences in power generation at wind speeds of 10 and 12 m/s, which are lower and higher than the rated wind speed, respectively, under different power levels. The results show that, at wind speeds of 10 and 12 m/s, the maximum power generation occurs at approximately 70% of the original power. When the wind speed is 10 m/s, the maximum power-generation point occurs at 71%; when the wind speed is 12 m/s, the maximum power-generation point occurs at 72%. However, at a wind speed of 12 m/s, power generation increases by only 7.03% compared with the original power generation. The plots in Figure 14 also indicate that, when variable-speed control is considered, wind speed has only a minor influence on the optimal life-extension approach for the power system. Regardless of wind speed, maximum power generation occurs when the power is approximately 70% of the original power.

To present the results more clearly, the RUL and power generation under variable-speed control are summarized in Table 6. The results indicate that, from the perspective of RUL, a lower WTG power level is more favorable. However, lower power does not necessarily result in better power generation.

**Figure 15:**
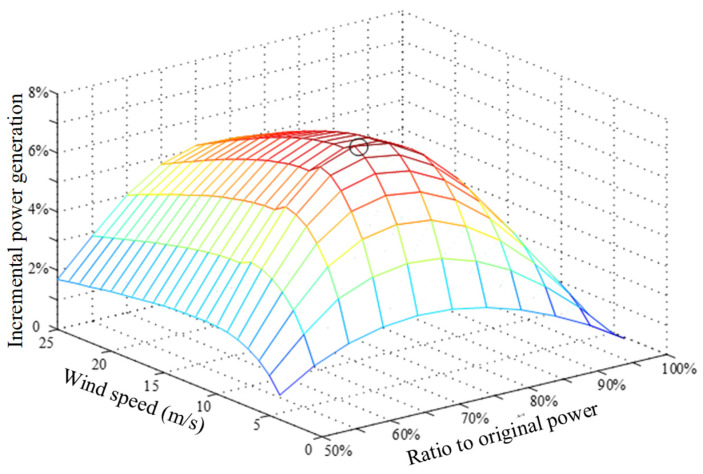
Power generation under pitch control.

2. Pitch control: The power generation results obtained using pitch control are shown in Figure 15. The results indicate that, across different power levels, the results are similar to those obtained with variable-speed control, and the maximum power generation of the WTG occurs at approximately 70% of the original power. Across different wind speeds, the results are also similar to those of variable-speed control, and maximum power generation occurs at a wind speed of 10 m/s.

**Figure 16:**
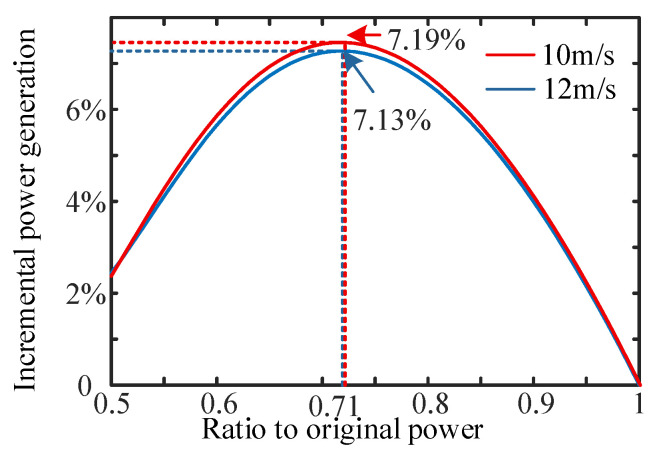
Power generation under pitch control at different power levels.

The variation in power generation at wind speeds of 10 and 12 m/s, which are below and above the rated wind speed, respectively, under different power conditions, is shown in Figure 16. The results indicate that the maximum power generation occurs at approximately 70% of the initial power. Specifically, at both 10 and 12 m/s, the maximum power generation corresponds to 71% of the original power. When the wind speed is 10 m/s, the power generation growth rate is 7.19% relative to the original power, whereas at 12 m/s, this growth rate is 7.13% relative to the original power.

Compared with variable-speed control, pitch control produces a greater increase in power generation. This is attributable to the fact that when the wind speed exceeds 10 m/s, the wind turbine operates at full power. Although the RUL of the WTG under pitch control is shorter, its average operating power is higher, which enables more power to be generated over a short period at high power levels.

In addition, Figure 16 indicate that, under pitch control, the influence of wind speed on the optimal life-extension strategy is relatively small. Across all wind speeds, maximum power generation consistently occurs when the power is approximately 70% of the original power generation.

To present the results more clearly, the RUL and power generation under the two control strategies are summarized in Table 7. Regarding the relationship between power, RUL, and electricity generation, the same conclusion can be drawn from Table 6 and Table 7 as in the case of variable-speed control. The only difference is that the increase in electricity generation varies under different operating conditions.

In this study, the optimal operating power of the WTG is approximately 70% of its nominal power. When the wind speed is lower than the rated value, variable-speed control is recommended. At 10 m/s, this strategy achieves a maximum power-generation increase of 7.3% relative to the nominal output. In contrast, when the wind speed exceeds the rated value, pitch control should be adopted.

### 3.4. Verification of RUL and Power Generation Results After Life Extension

**Figure 17:**
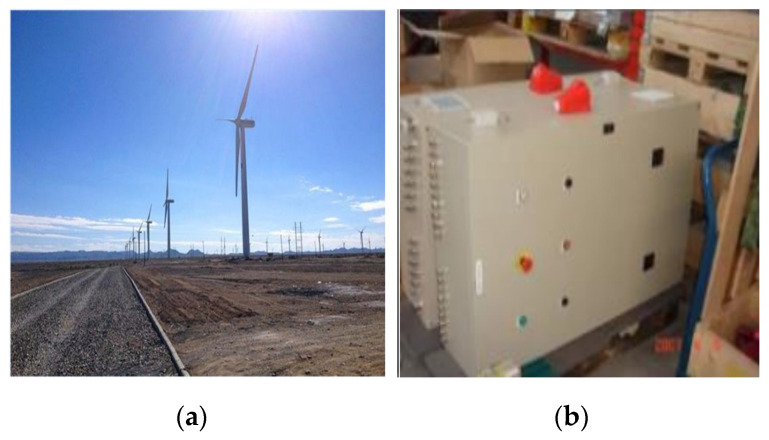
Wind farm and data acquisition device. (**a**) Wind farm; (**b**) Data acquisition device.

The results of this study were validated using actual data from two wind farms. The on-site photographs and data collection devices for one of the wind farms are shown in Figure 17.

**Figure 18:**
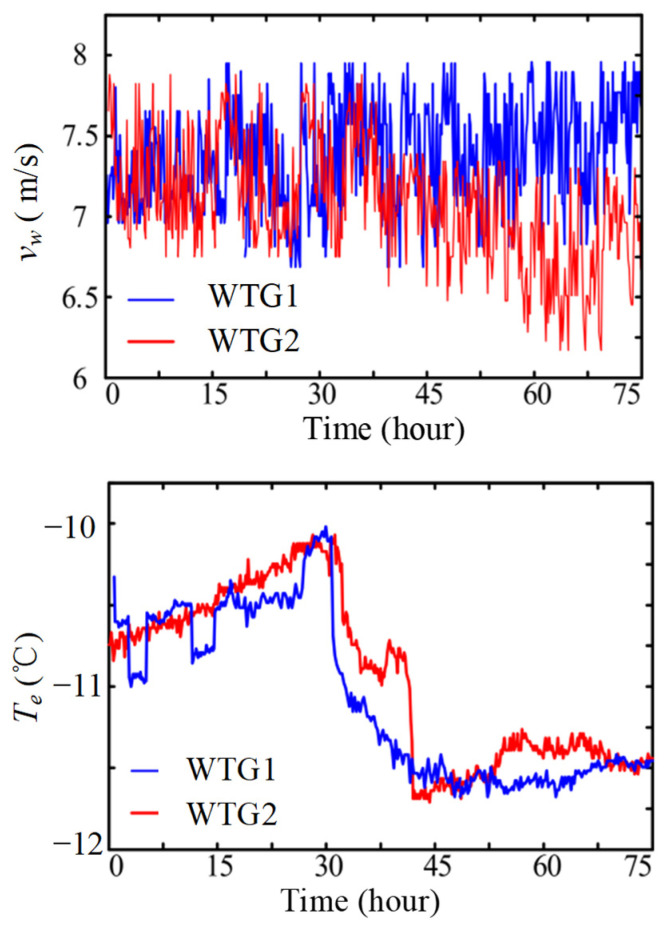
Environmental conditions of different wind turbine gearboxes.

We used two identical models of WTGs to verify the RUL and power generation of wind turbines after life extension under the same operating conditions and different power levels. The structural parameters of the two WTGs were identical. Their operating conditions are shown in Figure 18. It can be seen that the operating environments of the WTGs were generally the same, with wind speeds ranging from 6.5 m/s to 8 m/s. After 48 h, the wind speed of the second WTG decreased suddenly, but remained above 6 m/s. Additionally, the environmental temperatures during the two periods were also similar.

**Figure 19:**
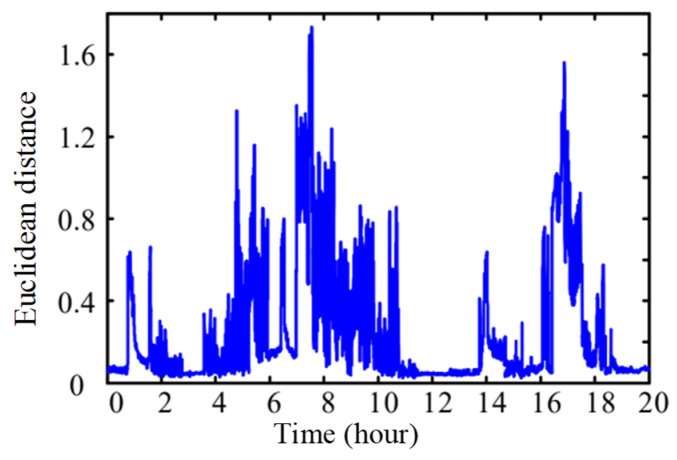
Average European distance of different wind turbine gearboxes.

Previous work [41] has demonstrated that Euclidean distance can be used to determine the similarity between two datasets. After calculation, the average Euclidean distance of each evaluation index for the two wind turbines is shown in Figure 19, with a standard deviation less than the statistical threshold of 2.06. Therefore, the operating status of the two WTGs can be compared during these two periods.

The Second WTG can be regarded as the First WTG under variable speed control, when the average wind speed is 8.3 m/s and the actual power is 75% of the original power. The calculated life-extension and power-generation increment according to the method proposed in this article are compared with the actual life-extension and power-generation increments in Table 8. As shown in Table 8, during time period 2, the RUL calculated according to the proposed method in this article was 67.6 h, corresponding to an accuracy rate of 93.2% compared with the actual RUL. The unit-time power-generation increment calculated according to the method in this article was 5.75% of the original power generation, corresponding to an accuracy of 87% compared with the actual power-generation increment. The above results provide evidence supporting the accuracy of the results in this article.

Based on the above method, we selected two additional sets of WTG s for verification, and the results obtained are shown in Table 8. The results show that the accuracy of the calculated RUL according to the method proposed in this paper exceeds 90% of the actual RUL. The accuracy of the power-generation increment calculated according to the method proposed in this article exceeds 85%. The above results demonstrate the effectiveness and accuracy of the proposed RUL extension strategy in extending the service life of WTGs.

**Figure 20:**
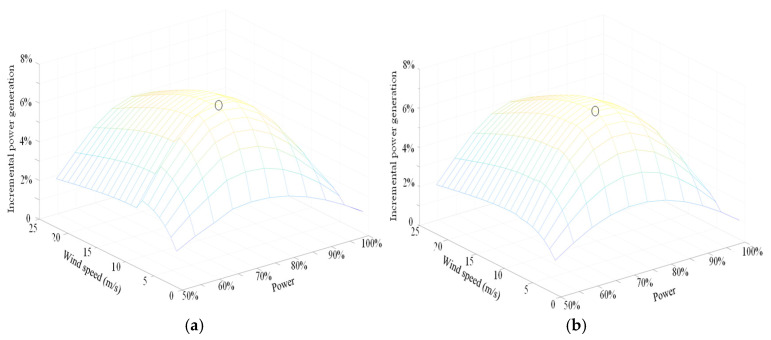
Power generation results for the second wind farm. (**a**) Variable-speed control; (**b**) Pitch control.

In addition, the proposed method was applied to calculate power generation after life extension for two other wind farms. The results for one of these wind farms are shown in Figure 20, which indicates that its power generation trend is similar to that of the first wind farm. The maximum increments in power generation and the corresponding operating conditions for the two additional wind farms are presented in Table 9 and Table 10. The operating conditions that produce maximum power generation in these two wind farms are consistent with those of the first wind farm. However, their maximum power generation increments are only 7.53% and 7.78%, respectively.

## 4. Conclusions

Not all wind turbine faults require immediate shutdown. Accurate assessment of WTG health status enables effective prediction of their RUL, which supports the development of a scientific life-extension strategy. This approach aims to improve operational efficiency, reduce operating costs, and increase revenue. In this study, the life-extension strategy for WTGs was developed by adjusting the rotational speed and pitch angle of the turbine, and the effects of two control strategies on RUL and power generation under different operating conditions were examined. The conclusions of this paper are summarized as follows:

1. The effects of two control strategies on the RUL of WTGs under different operating conditions were analyzed. The findings show that both strategies can achieve life extension, with variable-speed control demonstrating the strongest effectiveness.
2. The influence of the proposed WTG life-extension strategy on power generation was evaluated. The results demonstrate that this strategy can increase wind turbine power output, and the optimal control method varies depending on the operating conditions.

Future research can be conducted in the following areas:

1. Wind turbines in other regions can be analyzed to obtain more general conclusions.
2. Future studies may build on the research group’s existing foundation to expand the investigation to the entire transmission chain.
3. More accurate methodologies should be employed to determine the RUL of WTGs.

## Figures and Tables

**Table 1:** Correlation analysis results for selected monitoring indexes.

	*T_rb_*	*n*	*T*	*F_i_*	*F_o_*	*T_o_*
***T_rb_***	1	0.43	0.77	−0.19	0.002	0.97
***n***	0.43	1	0.84	0.31	0.48	0.47
***T***	0.77	0.84	1	0.10	0.28	0.80
***F_i_***	−0.19	0.31	0.10	1	0.94	−0.09
***F_o_***	0.002	0.48	0.28	0.94	1	0.11
***T_o_***	0.97	0.47	0.80	−0.09	0.11	1

**Table 2:** Evaluation indices for the wind turbine gearbox.

No.	Signal	Notation	Unit
**1**	Rear-end temperature of the high-speed shaft	*T_r_*	°C
**2**	Rear bearing temperature of the high-speed shaft	*T_rb_*	°C
**3**	Gearbox oil temperature	*T_o_*	°C
**4**	Output power	*P*	kW
**5**	Spindle speed	*n*	rpm
**6**	Generator inlet oil pressure	*F_i_*	bar

**Table 3:** Signals used for the prediction of evaluation indicators.

No.	Signal	Notation	Unit
**1**	Gearbox inlet oil temperature	*T_i_*	°C
**2**	Environment temperature	*T_e_*	°C
**3**	Wind speed	*v_w_*	m/s
**4**	Rotor speed	*v_r_*	rpm
**5**	Gearbox oil pump outlet pressure	*F_o_*	bar

**Table 4:** Root mean square values and kurtosis for actual and simulated signals.

Characteristic	Simulated *n*	Actual *n*	Simulated *T_o_*	Actual *T_o_*
**RMS**	1211.5	1213.2	42.89	42.85
**Kurtosis**	2.59	2.97	3.45	3.62

**Table 5:** Ranges of WTG evaluation indexes.

Signal	Lower Limit	Upper Limit
***P***	0	2200 kW
***n***	0	2000 rpm
***T_o_***	*T_e_*	85 °C
***T_r_***	*T_e_*	100 °C
***T_rb_***	*T_e_*	95 °C
***F_i_***	0.25 bar	0.8 bar

**Table 6:** RUL and power generation after life extension using variable-speed control.

Ratio to Original Power	Wind Speed (m/s)	Increase in RUL	Increase in Power Generation
**90%**	10	15.9%	3.8%
	12	16.1%	3.8%
**80%**	10	38.3%	6.4%
	12	33.2%	6.3%
**70%**	10	56.7%	7.3%
	12	59.1%	7.0%

**Table 7:** RUL and power generation after life extension using pitch control.

Ratio to Original Power	Wind Speed (m/s)	Increase in RUL	Increase in Power Generation
**90%**	10	14.4%	3.7%
	12	14.5%	3.9%
**80%**	10	31.2%	6.3%
	12	31.2%	6.5%
**70%**	10	52.8%	7.2%
	12	52.8%	7.1%

**Table 8:** Comparison of residual life and power generation in different cases.

Case	1	2	3
**Ratio of actual power**	75%	80%	90%
**Average wind speed (m/s)**	8.3	10.9	16.3
**Control strategy**	Variable-speed control	Variable-speed control	Pitch control
**Original lifespan (h)**	50	62	106
**Actual lifespan (h)**	63	73	112
**Calculate lifespan (h)**	67.6	75.3	122.3
**Actual incremental power generation**	5.01%	4.3%	3.7%
**Calculate the incremental power generation**	5.75%	4.7%	4.1%

**Table 9:** Power generation results for the second wind farm.

Control Strategy	Wind Speed Range (m/s)	Maximum Increase in Power Generation	Wind Speed (m/s)	Ratio to Original Power
**Variable-speed control**	*v_w_* < 10.8	7.42%	10	0.71
	10.8 < *v_w_*	7.33%	12	0.69
**Pitch control**	*v_w_* < 10.8	7.36%	10	0.7
	10.8 < *v_w_*	7.34%	10	0.72

**Table 10:** Power generation results for the third wind farm.

Control Strategy	Wind Speed Range (m/s)	Maximum Increase in Power Generation	Wind Speed (m/s)	Ratio to Original Power
**Variable-speed control**	*v_w_* < 10.8	7.15%	10	0.72
	10.8 < *v_w_*	7.01%	12	0.71
**Pitch control**	*v_w_* < 10.8	7.12%	10	0.68
	10.8 < *v_w_*	7.17%	10	0.69

## Data Availability

Due to the fact that the data is sourced from China Shipbuilding Group Haizhong Wind Power Co., Ltd. and is considered private data. The data presented in this study are available on request from Haizhong Wind Power Co., Ltd.
